# Extracellular RNA: mechanisms of secretion and potential functions

**DOI:** 10.1093/jxb/erac512

**Published:** 2023-01-04

**Authors:** M Lucía Borniego, Roger W Innes

**Affiliations:** Department of Biology, Indiana University, Bloomington, IN 47405, USA; Department of Biology, Indiana University, Bloomington, IN 47405, USA; Instituto de Agrobiotecnología del Litoral, Argentina

**Keywords:** Apoplast, plant–microbe interactions, RNAi, secretion, tasiRNA, tRNA fragments, trans-kingdom RNAi

## Abstract

Extracellular RNA (exRNA) has long been considered as cellular waste that plants can degrade and utilize to recycle nutrients. However, recent findings highlight the need to reconsider the biological significance of RNAs found outside of plant cells. A handful of studies suggest that the exRNA repertoire, which turns out to be an extremely heterogenous group of non-coding RNAs, comprises species as small as a dozen nucleotides to hundreds of nucleotides long. They are found mostly in free form or associated with RNA-binding proteins, while very few are found inside extracellular vesicles (EVs). Despite their low abundance, small RNAs associated with EVs have been a focus of exRNA research due to their putative role in mediating trans-kingdom RNAi. Therefore, non-vesicular exRNAs have remained completely under the radar until very recently. Here we summarize our current knowledge of the RNA species that constitute the extracellular RNAome and discuss mechanisms that could explain the diversity of exRNAs, focusing not only on the potential mechanisms involved in RNA secretion but also on post-release processing of exRNAs. We will also share our thoughts on the putative roles of vesicular and extravesicular exRNAs in plant–pathogen interactions, intercellular communication, and other physiological processes in plants.

## Teaser

In this review, we discuss the biological significance of extracellular RNAs in plants and provide insights into their secretion, processing, and trafficking within the apoplast.

## Introduction

The plant extracellular space, alternatively known as the apoplast, is a partially interconnected matrix external to the plasma membrane that includes the free space between cells (or intercellular space), xylem, cell walls, and apoplastic fluid. Many biological processes take place in this compartment, including nutrient exchange, cell wall biosynthesis, plant signaling, and defense responses (Sattelmacher and Horst, 2007; Floerl *et al.*, 2012; O’Leary *et al.*, 2016). To study its composition, the apoplast soluble fraction can be isolated by vacuum infiltration with an appropriate extraction buffer, followed by gentle centrifugation to collect the so-called apoplastic wash fluid (AWF) (Lohaus *et al.*, 2001). The composition of the AWF is modulated during plant development and in response to biotic interactions and abiotic stresses (López-Millán *et al.*, 2000; O’Leary *et al.*, 2016; Borniego *et al.*, 2020; Farvardin *et al.*, 2020). It contains molecules related to metabolism and signaling, nucleic acids, and diverse proteins, including a variety of proteases and nucleases (Sattelmacher and Horst, 2007; Guerra-Guimarães *et al.*, 2016; Borniego *et al.*, 2020).

**Fig. 1.**
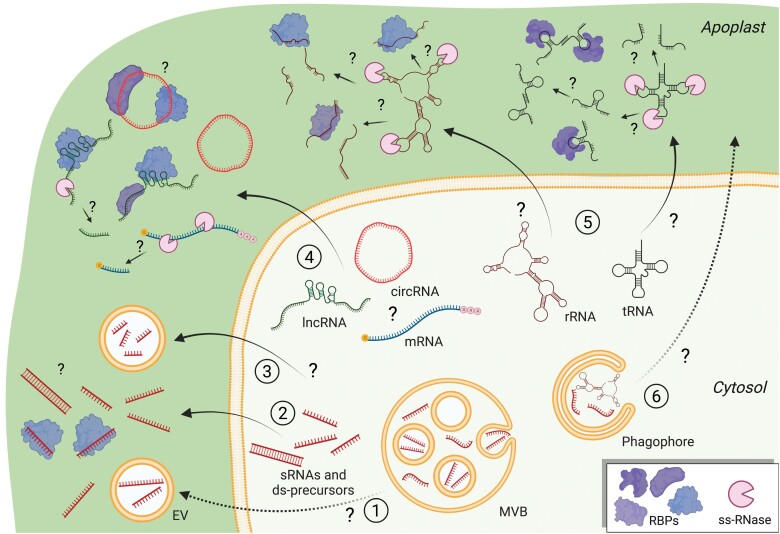
Classes of exRNAs and potential sources. It has been suggested that siRNAs and miRNAs can be secreted into the apoplast inside MVB-derived EVs (Cai *et al.*, 2018) (1). However, most of these sRNAs and possibly their double-stranded precursors are secreted via an EV-independent pathway and associate with RBPs in the apoplast (Zand Karimi *et al.*, 2022) (2). tyRNAs are preferentially secreted inside EVs and their biogenesis is still unknown (Baldrich *et al.*, 2019) (3). Many lncRNAs and circRNAs are well protected from RNase digestion in the apoplast, possibly through association with proteins and due to the presence of post-transcriptional modifications that prevent cleavage (Zand Karimi *et al.*, 2022). However, the existence of extracellular sRNA fragments derived from lncRNAs and from mRNAs suggests that these classes of RNAs are also prone to processing by extracellular RNases (4). rRNAs and tRNAs are probably secreted as full length and, once in the apoplast, they could be rapidly processed by extracellular RNases to generate rRFs and tRFs that acquire high RNase resistance possibly by association with proteins and/or by forming stable dimers (5). Cellular RNAs can be engulfed during autophagosome formation, although there is still no evidence of secretory autophagy in plants (6).

The presence of RNA in the plant apoplast was first suggested in the 1980s and 1990s with the discovery that plant cells secrete RNases (Nurnberger *et al.*, 1990; Oleson *et al.*, 1982). These extracellular enzymes were proposed to be involved in degrading extracellular RNA substrates for recycling inorganic phosphate (P_i_). Due to the presence of these RNases, RNA has generally been considered to be unstable in the apoplast, unless it is protected from RNase digestion, either by encapsulation within lipid membrane-containing extracellular vesicles (EVs) or by tight association with RNA-binding proteins (RBPs) (Koch and Wassenegger, 2021). The discovery that plant EVs carry defense-related proteins and small RNAs (sRNAs; here defined as all RNAs <35 nt in length) has led to the speculation that plant EVs are the key players in trafficking regulatory sRNAs throughout a plant and into invading pathogens (Rutter and Innes, 2017; Cai *et al.*, 2018; Baldrich *et al.*, 2019; Hou *et al.*, 2019). However, it has recently become evident that non-vesicular RNAs are the main constituents of the extracellular RNA (exRNA) pool in both plants and animals, despite receiving far less attention than their EV-associated counterparts (Baldrich *et al.*, 2019; Tosar *et al.*, 2020; Zand Karimi *et al.*, 2022). The plant exRNAome comprises a heterogenous group (summarized in Box 1 and Fig. 1) ranging from tiny RNAs (10–17 nt) to long non-coding RNAs (lncRNAs) of at least 1 kb in length (Mukherjee *et al.*, 2020; Zand Karimi *et al.*, 2022). Here, we review the diversity of exRNA species found in the plant apoplast, and then discuss the possible mechanisms involved in exRNA secretion, processing, and trafficking within the plant extracellular milieu. We will also address the potential functions of different classes of exRNA during plant development and biotic interactions.

## Methods used to quantify exRNA in plants. Getting a reliable assessment of the extracellular RNAome

Bulk analyses of the composition of exRNA have generally been accomplished through gel electrophoresis and next-generation RNA sequencing (Baldrich *et al.*, 2019; Zand Karimi *et al.*, 2022), while the detection and quantification of specific exRNA species have been conducted almost entirely using semi-quantitative (endpoint) reverse transcription–PCR (RT–PCR) methods (Cai *et al.*, 2018; He *et al.*, 2021). Technical limitations of all these methodologies can introduce biases in the representation of individual sequences. For instance, endpoint RT–PCR is a good tool for validating the presence of a given RNA sequence due to its high sensitivity, but its limited dynamic range makes it an unreliable tool for quantification (Bustin, 2000; Smith and Osborn, 2009). More accurate estimation of transcript abundances within the starting material can be achieved using reverse transcription–quantitative PCR (RT–qPCR; Smith and Osborn, 2009). Conversely, when using standard RNAseq methods, one must be aware that the RNA molecules detected are commonly biased toward the species most amenable to the library preparation methods. Modifications on RNAs may interfere with adapter ligation and reverse transcription during library preparation (Werner *et al.*, 2020; J. Wang *et al.*, 2021) and, consequently, highly modified non-coding RNAs (ncRNAs) such as tRNAs, rRNAs, and sRNAs derived from these RNA species are particularly difficult to sequence using standard procedures (X. Zhang *et al.*, 2016; Cognat *et al.*, 2017; Schimmel, 2018; Sergiev *et al.*, 2018). With the emergence of specialized library preparation procedures that have been developed to overcome these limitations (Cozen *et al.*, 2015; Zheng *et al.*, 2015; Honda *et al.*, 2016; Shi *et al.*, 2021; H. Wang *et al.*, 2021; Gu *et al.*, 2022), we anticipate that a more realistic picture of the exRNAome will emerge in the future through applying a combinatorial approach. To accurately assess the diversity of exRNAs, proper sequencing procedures should be used in combination with validation methods, such as northern blot analyses or RT–qPCR.

## Determining whether an RNA species is located inside or outside EVs

To elucidate how different RNA species are secreted and how they are trafficked between cells, it is critical to determine whether they are located inside or outside EVs. Apoplastic RNAs can be grouped into three general classes: intravesicular RNA (encapsulated inside EVs), particle-bound extravesicular RNA [located outside EVs, but associated with particles that can be pelleted by centrifugation at 100 000 *g* (P100)], and soluble RNAs that remain in the P100 supernatant. Separation of particle-bound extravesicular RNA from intravesicular RNA is challenging (Vickers *et al.*, 2011; Thery *et al.*, 2018; Jeppesen *et al.*, 2019). Plant EVs are usually isolated from AWF using differential ultracentrifugation (DUC) protocols (Regente *et al.*, 2009; Rutter and Innes, 2017); however, exRNAs circulating as ribonucleoprotein particles co-purify with EVs during standard DUC. While further purification of EV preparations using high-resolution density gradient centrifugation can remove some of the non-EV co-precipitants, it is still possible for some ribonucleoprotein complexes to co-purify with the EV-rich fraction (Sodar *et al.*, 2016). Therefore, regardless of the purification method used, to convincingly demonstrate that a given RNA species is located inside EVs, the non-vesicular RNAs should be eliminated before moving forward with sequencing or RT–qPCR analysis. RNase treatments can be used to eliminate extravesicular RNA; however, many exRNAs are resistant to degradation by RNases due to their association with RBPs (Arroyo *et al.*, 2011; Zand Karimi *et al.*, 2022). To be confident that exRNAs are located inside EVs, RNase protection assays should be performed using RNase alone, protease plus RNase, and detergent plus protease plus RNase. (Zand Karimi *et al.*, 2022). The protease plus RNase treatment should eliminate any RNA associated with proteins located outside EVs, while leaving RNAs located inside EVs intact (Arroyo *et al.*, 2011; Rutter and Innes, 2020; Zand Karimi *et al.*, 2022). When performing such assays, though, it is important to confirm that protease treatment alone does not disrupt the integrity of the EVs (Zand Karimi *et al.*, 2022). When assessing the location of specific exRNAs, we would further recommend that the abundance in the P100 supernatant (i.e. the soluble fraction) be quantified by RT–qPCR or RNAseq and compared with the P100 pellet. An intravesicular RNA should be enriched in the intravesicular fraction (P100 pellet) relative to the soluble fraction.

## The plant extracellular RNAome

### Regulatory sRNAs. Are they preferentially packaged inside EVs?

Cai *et al.* (2018) were the first to report the presence of specific regulatory sRNAs inside plant EVs. By means of semi-quantitative RT–PCR, the authors showed that the trans-acting siRNAs (tasiRNAs, TAS) TAS1c-siR483 and TAS2-siR453, as well as miRNA166 and IGN-siR1 co-purified with EVs labeled with the EV marker protein TETRASPANIN8 (TET8). Furthermore, these sRNAs were protected from degradation by micrococcal nuclease. Based on these observations, they concluded that these sRNAs were packaged inside EVs. In a follow-up study, three of these sRNAs were identified in multiple TET8-containing fractions collected from a sucrose density gradient, and also in TET8 particles that were isolated by immunoaffinity capture from crude EV pellets (He *et al.*, 2021), further establishing the association of these sRNAs with TET8-labeled EVs. Similarly, using RT–qPCR, Hou *et al.* (2019) were able to detect these siRNAs in Arabidopsis EVs purified using an iodixanol density gradient. However, the RNase protection assays used in these reports did not include a protease pre-treatment, and thus may not have eliminated RNAs protected from degradation by protein complexes. Interestingly, it has been shown that only a very low fraction of the miRNAs that mammalian cells secrete into the extracellular space are actually packaged inside EVs (Arroyo *et al.*, 2011; Turchinovich *et al.*, 2011; Albanese *et al.*, 2021).

Small RNA sequencing revealed that Arabidopsis EVs are enriched in tinyRNAs, a special class of very short (10–17 nt) sRNAs, while relatively few 18–34 nt sRNAs are packaged inside EVs (Baldrich *et al.*, 2019). Only seven miRNAs were found to be enriched inside EVs relative to outside EVs in AWF, six of which correspond to passenger strands of active miRNAs, suggesting that the miRNAs packaged inside EVs represent waste material that is being discarded from the cell. Additionally, the majority of EV-loaded tinyRNAs appear to be degradation products derived from multiple sources, such as transposable elements (TEs), mRNAs, intergenic regions, Pol4 precursors, rRNA regions, tRNAs, and miRNA precursors (Baldrich *et al.*, 2019; Zand Karimi *et al.*, 2022). Notably, in mammalian cells, specific tinyRNAs have been found to enhance the slicing capacity of human ARGONAUTE 3 (AGO3), while reducing AGO2 activity (Park *et al.*, 2020). Whether plant tinyRNAs have a regulatory role in silencing or just represent metabolic waste remains unknown. Also, we should consider that some regulatory sRNAs could be selectively packaged in EVs in response to pathogen infection or other types of stress. In plants, EV secretion is enhanced during biotic stress (Rutter and Innes, 2017), and it is plausible that the RNA content inside EVs also changes in response to stress, but this has not been demonstrated to date.

It has recently been shown that mammalian RNA-containing EVs are small (~50 nm) and relatively rare in a general EV population (Barman *et al.*, 2022). If plants produce a small subpopulation of RNA-enriched EVs, it could explain why RNAseq methods have failed to detect enrichment of specific siRNA species in bulk EV preparations (Baldrich *et al.*, 2019; Zand Karimi *et al.*, 2022), but more study is needed to test this hypothesis.

### Other sRNAs in the apoplast

The apoplast also contains sRNA fragments derived from multiple RNA species, mainly tRNAs, mRNAs, Pol IV precursors, TEs, and rRNAs (Baldrich *et al.*, 2019; Zand Karimi *et al.*, 2022). While some of these fragments co-purify with EVs following centrifugation at 40 000 *g*, RNase protection analyses indicated that the majority of these RNAs are located outside EVs and are associated with proteins (Baldrich *et al.*, 2019; Zand Karimi *et al.*, 2022). However, the majority of sRNA fragments are not pelleted by centrifugation at 100 000 *g*, indicating that they are associated with neither EVs nor large protein complexes (Zand Karimi *et al.*, 2022). This leads us to speculate that these RNAs are intrinsically resistant to extracellular RNases, raising the question of what makes them resistant to RNase degradation. Notably, tRNA and 5.8S rRNA-derived fragments seem to be the most abundant sRNA species in the apoplast (Baldrich *et al.*, 2019; Kusch *et al.*, 2022, Preprint). It has been shown that mammals also accumulate tRNA halves in the extracellular milieu (Tosar and Cayota, 2020). Interestingly, most of these mammalian tRNA halves achieve high stability against extracellular single-stranded RNases through forming RNA dimers and tetramers (Lyons *et al.*, 2017; Tosar *et al.*, 2018, 2020).

### Extracellular long non-coding RNAs

Data generated in our lab indicate that very few full-length mRNAs co-purify with EVs; instead, most reads correspond to fragments derived from rRNA and intergenic regions, and to a lesser extent from TEs (Zand Karimi *et al.*, 2022). Notably, many of the fragments that correspond to protein-coding genes include introns, although contamination with genomic DNA cannot be completely ruled out. These observations suggest that exRNAs are enriched in incompletely spliced or alternatively spliced RNAs (Zand Karimi *et al.*, 2022). RNAseq analyses have not shown abundant full-length tRNA sequences in the exRNAome, but this may be due to artifacts associated with library preparation methods, as denaturing polyacrylamide gel analysis reveals prominent bands in the 75–90 nt size range expected for full-length tRNA (Baldrich *et al.*, 2019; Zand Karimi *et al.*, 2022). Sequencing of full-length mature tRNAs is challenging when using conventional RNAseq methods due to the abundance of post-transcriptional modifications, some of which hamper reverse transcription or adapter ligation.

In addition to tRNA-derived fragments (tRFs), Arabidopsis exRNA contains thousands of circular RNAs (circRNAs; Zand Karimi *et al.*, 2022). Since the plant apoplast is enriched in endoribonucleases from the T2 family that should be capable of digesting circRNA structures (MacIntosh and Castanet, 2020), we speculate that these extracellular circRNAs are protected by association with RBPs. Consistent with this, they can be pelleted by centrifugation at 40 000 *g* and can be digested by RNase A following protease treatment (Zand Karimi *et al.*, 2022). Important questions that remain to be answered are whether circRNAs are enriched in the apoplast relative to cellular RNA and, if so, are they preferentially secreted, or are they simply more stable than linear RNAs?

## Mechanisms involved in RNA secretion into the apoplast

### Sorting of RNAs into EVs

To date, most studies on RNA secretion have focused on the packaging of RNA into EVs. Several RBPs have been implicated as potential intermediates in this process (Villarroya-Beltri *et al.*, 2013; Mateescu *et al.*, 2017; Temoche-Diaz *et al.*, 2019; Fabbiano *et al.*, 2020; He *et al.*, 2021). However, the co-isolation of RNA–protein complexes and multiple EV subpopulations has made it difficult to identify the ways through which different RNAs are exported. Recently, He *et al.* (2021) showed that ARGONAUTE1 (AGO1), the ANNEXINS ANN1 and ANN2, and the DEAD-box RNA HELICASES RH11 and RH37 bind to sRNAs enriched in Arabidopsis EVs. These RBPs co-localize with the EV marker TET8 and the multivesicular body (MVB) marker ARA6 inside the plant cells, as well as with TET8 in EV preparations, suggesting that these RPBs, along with their associated sRNAs, are packaged into MVB-derived EVs (He *et al.*, 2021). Based on protease protection assays, all of these proteins are located inside EVs. According to RNA-binding specificity, the authors proposed that AGO1, RH11, and RH37 are involved in selective loading of specific sRNAs into EVs, while ANN1 and ANN2, which bind non-specifically to RNA, possibly contribute to the stabilization of RNA molecules inside EVs. Consistent with this idea, mutation of *ANN1* and *ANN2* together appears to reduce the level of sRNAs in Arabidopsis EV preparations, at least as assessed by end-point PCR (He *et al.*, 2021). Interestingly, it has been suggested that human annexin2 (ANXA2) plays a role in the loading of miRNAs into EVs (Hagiwara *et al.*, 2015). However, other studies have shown that human ANXA2 does not co-purify with EVs that are thought to transport RNA (exosomes; Jeppesen *et al.*,2019), and thus the role of annexins in exRNA export remains unclear.

Despite accumulating evidence that RBPs play a role in packaging sRNAs into EVs, the mechanism(s) by which RBPs and their associated RNAs are trafficked to and selected for loading into newly forming EVs is still largely unknown. Leidal *et al.* (2020) found that LC3, an ATG8 ortholog that captures substrates for autophagy, promotes biogenesis of a subpopulation of MVB-derived EVs that are enriched in sRNAs and RBPs in mammals. They proposed a mechanism in which the lipidated, membrane-bound form of LC3 (LC3-II), located at the MVB limiting membrane, directly captures RBPs and packages them into intraluminal vesicles (ILVs), which are subsequently released as EVs via MVB fusion with the plasma membrane (Leidal *et al.*, 2020).

A more recent study in mammals has uncovered a different mechanism of exRNA secretion that involves endoplasmic reticulum (ER)–plasma membrane contact sites that tether the proteins VAP-A and Ceramide Transfer protein (CERT). This study concludes that VAP-A controls intraluminal filling of MVBs with LC3 and specific miRNAs; however, the role of RBPs in this process is still unclear (Barman *et al.*, 2022). One plausible model is that RBP–RNA complexes are specifically trafficked to sites of EV biogenesis. In support of this model, several RBPs that are found inside EVs in Arabidopsis, such as AGO1, RHs, and annexins, are also known to regulate RNA subcellular localization (Linder and Jankowsky, 2011; Bologna *et al.*, 2018; Monastyrskaya, 2018). Therefore, it will be important to assess whether RNAs bound to these RBPs in whole-cell lysate are also found inside EVs through RNA immunoprecipitation and sequencing analysis.

### EV-independent secretion of RNA

Recent observations showing that extravesicular RNAs constitute the majority of exRNA suggest that plants preferentially secrete sRNAs and lncRNAs into the apoplast via mechanisms that do not involve their packaging inside EVs (Zand Karimi *et al.*, 2022). Considering that a significant fraction of exRNA is associated with RBPs outside EVs, we speculate that RBPs play a role in EV-independent secretion of exRNAs. Many RBPs have been detected in the apoplast of Arabidopsis leaves, including ANN1, ANN2, RH11, RH37, GLYCINE-RICH PROTEIN 7 (GRP7), AGO1, and AGO2. All of these proteins can bind to sRNAs, and GRP7 and AGO2 can also bind to lncRNAs in both cell lysates and apoplastic fractions (He *et al.*, 2021; Zand Karimi *et al.*, 2022). Protease protection assays indicate that a significant fraction of each of these proteins is located outside EVs, with the majority of GRP7 and AGO2 being located in the non-EV fraction. Mutations in all these extracellular RBPs alter the apoplastic sRNA and/or lncRNA content (He *et al.*, 2021; Zand Karimi *et al.*, 2022).

Apart from being implicated in multiple aspects of RNA biosynthesis and processing (Cordin *et al.*, 2006; Hutvagner and Simard, 2008; Jarmoskaite and Russell, 2011; Koster *et al.*, 2014; Monastyrskaya, 2018), RBPs found in the apoplast may also be involved in RNA trafficking. For instance, DEAD-box RHs participate in the nuclear export of mRNA in plants (Gong *et al.*, 2005). In addition to its role in gene silencing, Arabidopsis AGO1 is also involved in the nucleo-cytoplasmic shuttling of miRNAs. Suppression of AGO1–miRNA association in the cytosol promotes cell to cell movement of miRNAs (Bologna *et al.*, 2018; Fan *et al.*, 2022). In addition, AGO1 accumulates at the ER and associates with the ER integral membrane protein ALTERED MERISTEM PROGRAM1 (AMP1) to inhibit the translation of target RNAs on the ER in plants (Brodersen *et al.*, 2012; Li *et al.*, 2013).

While human AGO2 has been implicated in binding and sorting miRNA into EVs (McKenzie *et al.*, 2016), extravesicular AGO2–miRNA complexes in human plasma suggest that AGO2 may also be involved in the secretion or stability of extravesicular miRNA (Arroyo *et al.*, 2011). In addition, density gradient analyses have revealed that extracellular pools of human AGO1, AGO2, AGO3, and AGO4 associate with non-vesicular fractions (Jeppesen *et al.*, 2019), further supporting a non-EV mechanism for secretion of these RBPs.

Arabidopsis GRP7 is involved in the export of mRNAs from the nucleus to the cytoplasm under cold stress conditions (Kim *et al.*, 2008). Apart from being found in the nucleus and cytoplasm, GRP7 also localizes to the plasma membrane and has been shown to be involved in cell to cell transport of siRNAs in plants. The C-terminal glycine-rich domain (GR) seems to be crucial for GRP7 movement between adjacent cells, hypothetically through association with plasmodesmata receptors (Alexandersson *et al.*, 2004; Yan *et al.*, 2020). The annexins constitute a family of widely distributed phospholipid-binding peripheral membrane proteins capable of translocating from water-soluble to membrane compartments in a Ca^2+^-dependent manner (Hajjar and Krishnan, 1999). Interestingly, both ANN1 and ANN2 have been implicated in Golgi-mediated exocytosis of newly synthesized plasma membrane and cell wall materials in plant cells (Carroll *et al.*, 1998; Clark *et al.*, 2005). In human cells, both annexin 1- and annexin 2-positive microvesicles (MVs) have been detected budding off directly from the plasma membrane through an MVB-independent pathway (Jeppesen *et al.*, 2019). The association of annexins and other apoplastic RBPs with the endomembrane system suggests that exocytosis may contribute to RNA secretion independent of exosomes.

In mammals, the autophagy machinery, functionally linked to degradation and recycling, is also involved in unconventional protein secretion and EV biogenesis, as well as in RBP and RNA release into the extracellular space (Dupont *et al.*, 2011; Bel *et al.*, 2017; Leidal *et al.*, 2020). It is not clear, however, whether RNA secreted in an autophagy-dependent manner is packaged inside classic exosomes (i.e. EVs containing the tetraspanin CD63), or are extravesicular (Frankel *et al.*, 2017; Jeppesen *et al.*, 2019), though we guess that it is likely to be a combination of both. Since RNA resides in most cellular compartments, it is reasonable to assume that RNAs can also be engulfed during autophagosome formation during bulk and selective autophagy in plants (Floyd *et al.*, 2015; Michaeli *et al.*, 2016; Wu *et al.*, 2021). Indeed, rRNA has been shown to accumulate in autophagosomes and vacuoles of Arabidopsis when the RNase RNS2 is mutated, and the vacuolar accumulation is blocked by mutation of the *AUTOPHAGY5* gene (Floyd *et al.*, 2015). However, whether secretory autophagy also occurs in plants is not yet known (Zarsky, 2022). Additionally, dying cells could potentially be another source of extravesicular exRNAs.

### Potential role of post-transcriptional modifications in marking RNA for export

In plants, both small and long exRNAs are highly enriched in the post-transcriptional modification *N*^6^-methyladenosine (m^6^A) relative to total cellular RNA (Zand Karimi *et al.*, 2022). This enrichment is striking and suggests that the m^6^A modification plays a key role in exRNA secretion or stabilization. Stabilizing effects have been reported for the m^6^A mark in Arabidopsis (Anderson *et al.*, 2018). In addition, according to the mammalian literature, we speculate that it can also be involved in RNA trafficking and secretion. In HeLa cell cultures, the nuclear m^6^A ‘reader’ protein YTHDC1 mediates the transport of m^6^A-modified mRNAs to the cytoplasm via association with the adaptor protein SRSF3 and the nuclear mRNA export receptor NXF1 (Roundtree *et al.*, 2017). Apart from YTH domain proteins, other RBPs have been described as m^6^A readers in mammals. Of particular interest, the human hnRNPA2B1 protein functions as a nuclear m^6^A reader that, apart from being involved in processing of primary miRNAs, also controls the loading of specific miRNAs into EVs (Villarroya-Beltri *et al.*, 2013; Jiang and Ogretmen, 2014). Notably, the glycine-rich RBP GRP7, found in the non-EV fraction of the apoplast, has homology to hnRNPA2B1, suggesting that GRP7 may be performing similar roles in plants. However, to date, no plant RBPs identified in the apoplast have been shown to bind to m^6^A-modified RNA. Whether m^6^A modification plays a role in the secretion of exRNAs into the apoplast and/or contributes to their stability thus requires further investigation.

## Trafficking of exRNA

Current knowledge on RNA movement within the plant body comes mainly from studies on the spread of RNAi signals. It has long been known that regulatory sRNAs (siRNAs and miRNAs) move via the symplastic pathway, in which plasmodesmata along with the phloem establish a cytoplasmic network that links virtually all the cells of a plant (Maizel *et al.*, 2020; Yan and Ham, 2022). Apart from sRNAs, other RNA species, including mRNAs, rRNAs, tRNAs, and tRFs, can move via the symplastic pathway, and several RBPs have been found to assist during this process (Liu and Chen, 2018). It is generally thought that movement of RNAs, especially long RNAs, through the apoplast is challenging because of the presence of cell walls. Although its permeability is modified by developmental and environmental factors, it is commonly assumed that molecules larger than 20 kDa do not move freely through plant cell walls (Guerra-Guimarães *et al.*, 2016), and many exRNAs are larger than that. Moreover, as the cell wall is normally negatively charged, the movement of charged molecules is affected by electrostatic interactions (Sattelmacher, 2001). However, there is some evidence supporting long-distance movement of RNA through the apoplastic pathway. For instance, recently Brosnan *et al.* (2021, Preprint) reported that high levels of intact and unprocessed 350 nt dsRNA were detected in roots and shoot apex 24 h after dsRNA application in rosette leaves of Arabidopsis plants in which symplastic transport had been blocked by induction of callose deposition. Indeed, they were able to detect dsRNA in apoplastic fluids, but not in extracellular vesicles, in both the leaves to which dsRNA had been applied and distal tissues. Surprisingly, the apoplastic pool of dsRNA was translocated to newly formed tissues 2 weeks post-application of the dsRNA to the source leaves, suggesting that dsRNAs are quite stable in the apoplast (Brosnan *et al.*, 2021, Preprint). Similarly, 22 nt siRNA and 500 nt hairpin (hp) RNA applied directly to the vasculature by petiole absorption at basal leaf positions were transported systemically to apical leaves in several plant species (Dalakouras *et al.*, 2018). According to this study, the siRNAs and long hpRNAs were trafficked as unprocessed molecules exclusively via the apoplastic pathway.

RNA mobility through the apoplast might be facilitated by association with RBPs. In agreement with this, it has been shown that lysine-containing cell-penetrating peptides help RNAs to cross cell walls and penetrate plant cells (Numata *et al.*, 2014). Also, certain RNA structure motifs that are common in exRNAs, including tRNA-like structures, are involved in RNA transport to distal tissues (T. Wang *et al.*, 2021). For instance, W. Zhang *et al.* (2016) demonstrated that fusion of some tRNA-like structures with immobile mRNAs can make the transcripts mobile, while removal of the tRNA motif from the mRNA–tRNA transcript disrupts the mobility. Remarkably, the mRNA–tRNA transcripts can be translated into functional proteins after being transported to distal tissues (W. Zhang *et al.*, 2016). In agreement, many phloem-mobile mRNAs contain tRNA sequences in their untranslated regions (UTRs) (Tolstyko *et al.*, 2020a), and 27% of the RNA fragments moving through the phloem sap in pumpkin have been shown to correspond to specific tRNAs and tRNA-derived halves (Zhang *et al.*, 2009). Based on these data, we can speculate that extracellular tRFs can assist movement of other classes of RNA within the apoplast.

The mechanism by which tRNA motifs induce movement of otherwise immobile mRNAs is thought to involve motif recognition by RBPs (W. Zhang *et al.*, 2016; Tolstyko *et al.*, 2020b). Interestingly, the mobility of tRNAs has also been correlated with the presence of methylated cytosine residues (5-methylcytosine, m^5^C). For instance, tRNA motifs that have a high percentage of m^5^C (tRNA^Gly^ or tRNA^Met^) can trigger mRNA mobility, while mRNAs fused with a tRNA motif that has a low level of m^5^C (tRNA^Ile^) are immobile (W. Zhang *et al.*, 2016; T. Wang *et al.*, 2021). Recent studies have shown that m^5^C promotes mRNA transport within the phloem, possibly through recognition and binding to specific RBPs (Yang *et al.*, 2019; Tolstyko *et al.*, 2020a). Notably, tRNA halves derived from tRNA^Gly^ and tRNA^Glu^ are especially abundant in Arabidopsis exRNA (Baldrich *et al.*, 2019). These tRNAs are known to be enriched in m^5^C (Burgess *et al.*, 2015; Baldrich *et al.*, 2019). Whether m^5^C modification is also involved in RNA movement in the apoplast remains to be elucidated.

The observation that plant EVs can deliver sRNAs into fungal pathogens (Cai *et al.*, 2018) raises the question of whether EVs are also capable of trafficking RNA between plant cells. However, experimental evidence for such trafficking is currently lacking and requires further investigation.

## Role of extracellular RNases in shaping the exRNAome

It is now evident that the vast majority of plant exRNAs are not associated with EVs. This implies that many exRNAs are exposed to degradation by extracellular RNases. In ­mammals, increasing evidence suggest that extracellular processing of RNAs is common. Two recent publications showed that mammalian cells secrete full-length tRNAs and rRNAs (associated with ribosomes) to the extracellular space, where they are processed by extracellular RNases (Nechooshtan *et al.*, 2020; Tosar and Cayota, 2020). Interestingly, inhibition of extracellular RNases in cultured mammalian cells leads to an exRNA banding pattern that reflects the intracellular RNA pool, suggesting that exRNAs, at least in cell culture media, may be released from dying cells (Tosar and Cayota, 2020) and are subsequently degraded by extracellular RNases. Regardless of the mechanism of RNA release, this finding indicates that exRNA processing plays a major role in shaping the exRNAome in mammals (Tosar and Cayota, 2020). This may also be true in plants. For instance, denaturing PAGE revealed that the exRNA pool of Arabidopsis leaves is characterized by an accumulation of <70 nt sRNAs that are completely absent in total RNA fractions, while the most abundant long RNAs in total RNA fractions are missing in extracellular fractions (Zand Karimi *et al.*, 2022). This pattern could indicate selective secretion of exRNAs, but is also consistent with extracellular processing of secreted RNAs.

Extracellular processing of RNA in plants is likely because plants secrete multiple RNases into the apoplast. These RNases belong to the T2 family (MacIntosh and Castandet, 2020). The members of this family are widely distributed among eukaryotic organisms, and they are also present in viruses and bacteria (Luhtala and Parker, 2010). All T2 RNases are targeted to the secretory pathway, therefore they are typically secreted from the cell or localize to intracellular organelles such as the ER, lysosomes, or the vacuole (Irie, 1999). The members of this family are non-specific endoribonucleases that cleave ssRNA in a two-step reaction that involves a first step of transphosphorylation that produces a 2ʹ,3ʹ cyclic phosphate (cP) intermediate, followed by a second step where these 2ʹ,3ʹ cyclic intermediates are hydrolyzed to generate mono- or oligonucleotides with a terminal 3ʹ-phosphate group and a 5ʹ-terminal hydroxyl at the cleavage site (Luhtala and Parker, 2010). Plant extracellular T2 RNases characterized so far catalyze the transphosphorylation step at a much faster rate than the hydrolysis of 2ʹ,3ʹ-cP intermediates. Consequently, RNA degradation by plant T2 RNases leads to accumulation of 2ʹ,3ʹ-cP intermediates (Abel et al., 1989, 2000; Nurnberger *et al.*, 1990; Gu *et al.*, 2022). These 2ʹ,3ʹ-cP intermediates as well as the final products following T2 cleavage are commonly missing from transcriptome analyses because standard RNAseq methods are unable to capture them (Shigematsu *et al.*, 2018). A better coverage of RNAs that have been processed by T2 enzymes can be achieved using specific sequencing methods, such as RtcB-sRNAseq or cP-RNAseq (Honda *et al.*, 2016; Nechooshtan *et al.*, 2020; Gu *et al.*, 2022).

Plant T2 enzymes are induced during development and in response to biotic stress and phosphate starvation (MacIntosh and Castandet, 2020). They have long been linked to P_i_ recycling (Bariola *et al.*, 1994) and are involved in rRNA turnover and tRNA processing (Hillwig *et al.*, 2011; Alves *et al.*, 2017; Megel *et al.*, 2019). It is assumed that degradation of rRNA by T2 enzymes takes place mostly in the vacuole by means of RNS2 (Floyd *et al.*, 2015, 2017), but we cannot rule out additional processing in the apoplast. In fact, many rRNA fragments accumulate in the apoplast of Arabidopsis leaves (Baldrich *et al.*, 2019; Zand Karimi *et al.*, 2022). Both vacuolar and secreted T2 RNases are essential for the production of tRFs through fragmentation of mature tRNAs at the single-stranded loop regions (Alves *et al.*, 2017; Megel *et al.*, 2019; Gu *et al.*, 2022). The subcellular localization where tRNA processing takes place is still unknown, though given that most RNase T2 enzymes are secreted from the cell, it is likely that tRF production takes place mainly in the apoplast. In agreement, a diversity of tRNA-derived halves preferentially accumulate in the non-vesicular extracellular fraction instead of inside cells in Arabidopsis leaves (Baldrich *et al.*, 2019; Zand Karimi *et al.*, 2022).

Like in Arabidopsis, tRNA^Glu^ and tRNA^Gly^ halves are also highly enriched in exRNA fractions of diverse mammalian cell lines as well as in a variety of human biofluids (Wei *et al.*, 2017; Srinivasan *et al.*, 2019; Nechooshtan *et al.*, 2020; Tosar and Cayota, 2020). Interestingly, these and other tRNA halves that are commonly found in the leaf apoplast can acquire high resistance to single-stranded RNases by forming self-protecting dimers or oligomers (Lyons *et al.*, 2017; Tosar *et al.*, 2018). This has led to the speculation that the accumulation of these sRNA species in the extracellular milieu is due to their high stability rather than to specific secretion (Tosar *et al.*, 2020). In addition to oligomerization-acquired stability, another layer of resistance to RNase degradation might be provided by the presence of methylation (Schaefer *et al.*, 2010). As noted above, there is a high correlation between the abundance of different classes of tRNA halves in the leaf apoplast and the degree of m^5^C modification, with highly methylated tRNA halves showing the highest accumulation and less methylated tRNAs under-represented in the non-vesicular fraction (Burgess *et al.*, 2015; Baldrich *et al.*, 2019). Although further investigation is required, these studies provide compelling evidence for post-release shaping of the exRNAome by extracellular RNases in plants.

## Potential functions of exRNAs

### P _i_ recycling

Inorganic phosphorus (P_i_) is an essential macronutrient for plants. Since the availability of P_i_ is usually low in soils, recycling and remobilization of P_i_ within the plant are crucial mechanisms that support plant growth and development. Nucleic acids are an important source of phosphate that plants can use to recycle P_i_. Many RNases, along with phosphatases and phosphodiesterases, are involved in the release of P_i_ from RNA to facilitate its remobilization (Goldstein *et al.*, 1989; Löffler *et al.*, 1992). Extracellular T2 RNases are highly induced during specific P_i_ starvation as well as during leaf senescence, a process that enables plants to recover nutrients from old tissues and re-use them in developing organs (Bariola *et al.*, 1994; Abel *et al.*, 2000; Tran and Plaxton, 2008; Borniego *et al.*, 2020). This indicates that exRNAs could play a significant role in P_i_ remobilization. In fact, it has been suggested that the extracellular P_i_ concentration rather than the intracellular P_i_ concentration elicits the induction of RNase activities in tomato (Glund and Goldstein, 1993).

ExRNAs may also represent an important source of P_i_ for plant pathogens. It has recently been shown that *Ustilago maydis* secretes T2 RNases into the plant apoplast during infection. These RNases can fully degrade naked RNA isolated from maize apoplastic fluids and the resulting nucleotides can be taken up by the fungus. Moreover, the absence of these enzymes was associated with reduced virulence and delayed fungus development due to the inability of the mutant fungus to utilize RNA as a source of phosphate (Mukherjee *et al.*, 2020).

### Plant–pathogen interactions

**Fig. 2.**
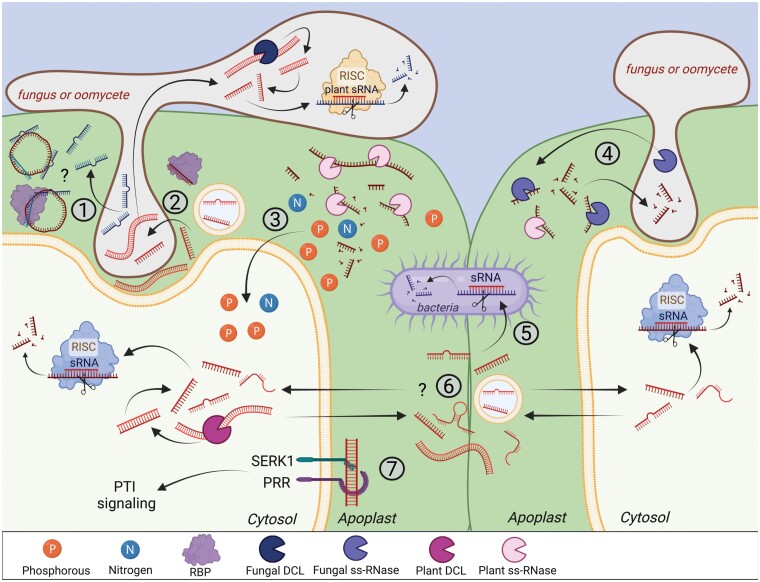
Potential roles of exRNAs. While the function of extracellular circRNAs in plants is unknown, the mammalian literature indicates that they can act as sponges to sequester miRNAs released by invading pathogens (Hansen *et al.*, 2013) (1). Naked sRNAs, long dsRNAs, and possibly EV-associated sRNAs can be taken up by pathogens directly from the apoplast and trigger silencing of pathogen target genes (Cai *et al.* 2018; Qiao *et al.* 2021). Long dsRNA is processed by fungal DCLs to generate sRNAs (Lax *et al.*, 2020) (2). exRNAs can serve as a source of nutrients, especially phosphorus (P) and nitrogen (N), that plant cells can reuse. For instance, during P_i_ starvation, many RNases along with phosphatases and phosphodiesterases are secreted into the apoplast to recover P_i_ from RNAs and support plant growth (Bariola *et al.*, 1994; Borniego *et al.*, 2020) (3). Some fungal pathogens secrete RNases into the plant apoplast that can completely digest plant exRNAs. The resulting nucleotides are taken up by the pathogen for use as a phosphate source to maintain fungal growth during infection (Mukherjee *et al.*, 2020) (4). Plants can send naked siRNA or EV-associated siRNAs into bacterial pathogens to silence virulence factors and reduce pathogenesis (Singla-Rastogi *et al.*, 2019) (5). sRNAs and long dsRNAs secreted by plants may be transferred between adjacent cells to regulate gene expression. Also, EVs could act as RNA carriers during cell–cell communication (6). Extracellular dsRNAs secreted by pathogens can be recognized as molecular patterns by membrane-associated receptor complexes and initiate PTI signaling responses within the cell (Niehl *et al.*, 2016) (7).

Given that the apoplast is a primary location for plant–pathogen interaction, it has been speculated that exRNAs may play a key role in this process. One proposed role for extracellular sRNAs is in trans-kingdom gene silencing, where specific sRNAs move between hosts and interacting organisms to silence genes (Fig. 2). An example of this process has been provided by Cai *et al.* (2018), who proposed that Arabidopsis cells secrete EVs containing specific sRNAs. Upon *Botrytis cinerea* infection, EVs accumulate at the infection sites and sRNAs are taken up by fungal cells where the transferred sRNAs inhibit fungal infection by targeting genes essential for pathogenicity (Cai *et al.*, 2018). It has long been speculated that EVs mediate delivery of regulatory sRNAs from plant cells to invading pathogens, since EVs provide an RNase-free environment where RNAs are protected from degradation. Recently, the group of Aline Koch has suggested that plants EVs may only play a minor or indirect role in the delivery and uptake of host‐induced gene silencing (HIGS)- and spray‐induced gene silencing (SIGS)-associated RNAs. They showed that co-cultivation of *Fusarium graminearum* (*Fg*) with EVs (P100) isolated from CYP3RNA-expressing *A. thaliana* plants as well as from CYP3RNA-sprayed barley plants did not have any effect on the expression of CYP51 in *Fg* (Schlemmer *et al.*, 2022). Interestingly, they had previously shown that >70% of HIGS-derived siRNAs in Arabidopsis were found to be extravesicular (Schlemmer *et al.*, 2021). These data are supported by several reports showing that although some extracellular siRNAs and miRNAs can be encapsulated inside EVs, most appear to be extravesicular (Cai *et al.*, 2018; Baldrich *et al.*, 2019; Zand Karimi *et al.*, 2022). These sRNAs could potentially be taken up by pathogens during infection. Some reports suggest that both long and short dsRNAs are highly stable in the plant apoplast (Dalakouras *et al.*, 2018; Brosnan *et al.*, 2021, Preprint). Consistent with this, no extracellular RNases capable of cleaving dsRNA have been identified in plants (MacIntosh and Castandet, 2020). It has been shown that several plant pathogens can take up short and long ss- and dsRNAs directly from the environment (M. Wang *et al.*, 2016; Qiao *et al.*, 2021; Cheng *et al.*, 2022), probably by the endocytic pathway (Wytinck *et al.*, 2020a, b). This suggests that extravesicular sRNAs could also undergo cross-kingdom trafficking. For instance, exogenously applied short and long dsRNAs that move exclusively through the apoplast were unable to trigger silencing of target genes in plants, but they effectively silenced genes of invading pathogens (Dalakouras *et al.*, 2018; Brosnan *et al.*, 2021, Preprint). Also, sRNAs can associate with AGO proteins in the apoplast (Brosnan *et al.*, 2021, Preprint; Zand Karimi *et al.*, 2022), and the secretion of AGO proteins into the apoplast increases upon pathogen infection (Brosnan *et al.*, 2021, Preprint). This has led to speculation that extravesicular RNA–protein complexes may be the key players in trans-kingdom gene silencing (Zand Karimi *et al.*, 2022). However, it is still unclear whether these RNA–protein complexes are functional inside pathogens, or whether silencing of pathogen genes still requires the canonical RNAi machinery of the pathogen.

Emerging evidence indicates that plant exRNAs may impact gene expression in bacterial pathogens through direct RNA:RNA base pairing. A preprint from 2019 reported that expression of hpRNAs in plants can silence homologous genes in the bacterial pathogen *Pseudomonas syringae*, which colonizes the leaf apoplast (Singla-Rastogi *et al.*, 2019, Preprint). This observation indicates that bacteria can potentially take up silencing RNAs that are secreted into the apoplast. Notably, this study also demonstrated that exogenous application of naked RNAs purified from Arabidopsis leaves expressing a hpRNA to axenically grown *P. syringae* efficiently silenced homologous genes, indicating that uptake of RNA by *P. syringae* does not require packaging into vesicles or protein complexes. This study also showed that silencing of target genes in *P. syringae* by Arabidopsis required the function of the Arabidopsis DICER-like proteins (DCLs) DCL2, 3, and 4 genes, which strongly suggests that siRNAs, rather than long hpRNAs, mediate gene silencing in this phyto-pathosystem. Collectively, these data suggest that plants secrete siRNAs into their apoplast where these RNAs can then impact the bacterial microbiome.

A more recent preprint provides further support for this hypothesis, reporting that Arabidopsis secretes sRNAs into the rhizosphere that were then taken up by root-associated bacteria (Middleton *et al.*, 2022, Preprint). That this could have functional consequences was supported by the finding that Arabidopsis mutants deficient in sRNA biogenesis were found to have dramatically altered root microbiomes. This effect, though, could be an indirect consequence of altered physiology in these mutants.

The expression of extracellular RNase T2 in response to stress correlates with the accumulation of tRFs (Alves *et al.*, 2017; Megel *et al.*, 2019), suggesting a regulatory role for extracellularly produced tRFs in this process. Like siRNAs and miRNAs, tRFs are also loaded into AGO proteins and regulate gene expression in plants, oomycetes, and animals (Loss-Morais *et al.*, 2013; Kumar *et al.*, 2014; Q. Wang *et al.*, 2016; Alves *et al.*, 2017; Ren *et al.*, 2019). For instance, tRFs have been shown to mediate trans-kingdom gene silencing between Rhizobia and soybean. Rhizobial tRFs produced by proccesing of tRNA^Gly^, tRNA^Gln^, and tRNA^Val^ associate with soybean AGO1 to catalyze tRF-guided cleavage of target mRNAs in soybean to promote nodulation (Ren *et al.*, 2019). Although direct evidence of a role for plant extracellular tRFs in cross-kingdom gene silencing is lacking, the high accumulation of tRFs in the leaf apoplast tempts us to speculate that this is likely. Thus, many classes of extracellular sRNAs, including siRNAs, miRNAs, and tRFs, in association or not with EVs or RBPs appear to be quite stable in the plant apoplast and could be taken up by invading pathogens to trigger silencing of virulence genes. In this context, it should be noted that the three core ­components of the eukaryotic RNA interfence pathway (RNA-dependent RNA polymerases, Dicers, and Argonautes) have been identified in the major groups of plant pathogenic fungi, including ascomycetes, basidiomycetyes, and zygomycetes (Lax *et al.*, 2020).

In addition to sRNAs, extracellular lncRNAs may also play a role in plant–pathogen interactions. CircRNAs, which are a subclass of lncRNAs, are induced by pathogen infection in plants and have been shown to contribute to defense against fungal infection (Fan *et al.*, 2020). RNAseq analyses revealed that the Arabidopsis leaf apoplast contains thousands of circRNAs, whose levels are modulated by AGO2 and GRP7, both of which have previously been shown to function in plant defense responses (Zand Karimi *et al.*, 2022). Although no function has been reported for any lncRNA in the plant apoplast, it has been speculated that apoplastic circRNAs may serve as sponges to sequester sRNA effectors secreted by pathogens, preventing sRNAs from reaching their target mRNAs inside host cells (Zand Karimi *et al.*, 2022) (Fig. 2). This putative role is supported by observations that oomycete and fungal pathogens produce sRNAs that target plant host genes and contribute to virulence (Dunker *et al.*, 2020; Weiberg *et al.*, 2013). Additional support for this sponge hypothesis comes from a study in mammals that showed that a circRNA named ciRS-7, which is conserved across eutherian mammals, acts as a sponge for the miRNA miR-7 (Hansen *et al.*, 2013). This circRNA strongly suppresses miR-7 activity, resulting in increased levels of miR-7 targets. Notably, ciRS-7 contains >70 strong binding sites for miR-7 that are highly conserved. Another example of a circRNA acting as an miRNA sponge is the oncogenic circCCDC66 (Hsiao *et al.*, 2017). Unlike ciRS-7 which carries numerous target sites for a single miRNA, circCCDC66 has multiple binding sites for different miRNAs and may sponge several miRNAs that target oncogenes.

### Intercellular communication

It has been reported that mammalian extracellular mRNA and miRNAs can be transferred into distant cells to regulate gene expression (Valadi *et al.*, 2007; Thomou *et al.*, 2017). A more recent study, however, reported that miRNAs delivered by mammalian EVs are non-functional in recipient cells, suggesting that EV-associated miRNAs do not play a significant role in cell to cell communication in mammals (Albanese *et al.*, 2021). Whether plants can employ exRNAs in intercellular and systemic communication is still unknown; however, recent findings led us to speculate that this may be possible. For instance, Gu *et al.* (2022) showed that two Arabidopsis extracellular RNases T2, RNS1 and RNS3, are induced upon *B. cinerea* infection and trigger fragmentation of many tRNAs. One of the tRFs produced by RNS1 and RNS3, 5ʹ tsR-Ala, associates with AGO1 and directs mRNA cleavage of the Arabidopsis gene *CYP71A13* to negatively regulate defense against *B. cinerea* (Gu *et al.*, 2022). The subcellular localization where 5ʹ tsR-Ala is produced is unknown. However, given that extracellular RNases, but not intracellular RNases, are essential for 5ʹ tsR-Ala production (Gu *et al.*, 2022), this tRF is probably produced in the extracellular space and subsequently taken up by plant cells to regulate gene expression.

Further evidence that plant cells can take up RNAs from the apoplast is provided by a recent report published by Shine *et al.* (2022), in which they were investigating the mobile signal responsible for systemic acquired resistance (SAR) in Arabidopsis, concluding that tasi-RNAs are the primary mobile signal. In that work, the authors infiltrated an *in vitro* synthesized *TAS3a* transcript (ss-T-555 RNA) into Arabidopsis leaves, which led to an increase in tasi-RNAs derived from this transcript in both local and distal tissues. Mutation of AGO7, a protein necessary for tasi-RNA biogenesis from *TAS3a*, abolished this increase in tasi-RNAs. These observations indicate that naked exRNA can be taken up by plant cells and processed by the endogenous RNAi machinery, although we cannot rule out that cell damage caused during the infiltration process could have facilitated T-555 cellular uptake. Also in this study, it was found that injection of 6-carboxyfluorescein (FAM)-labeled tasi-RNA into a leaf resulted in movement of the labeled RNA into the vasculature and movement into distal tissues within 3 h. Remarkably, the FAM–tasiRNA could be recovered intact from distal leaves, indicating that it was protected against degradation during systemic movement. This systemic movement appeared to be mediated by phloem transport, rather than apoplastic transport, as it could be blocked by overexpression of PDLP5, which inhibits movement through plasmodesmata (Shine *et al.*, 2022). As entry into the phloem from the apoplast requires uptake by cells, these observations indicate that tasi-RNAs are rapidly taken up by cells in the injected leaf.

Further support for RNA uptake from the apoplast comes from two studies examining induction of RNAi by spray application of dsRNAs to the tip of barley leaves. In these studies, the applied dsRNAs entered through stomata into the plant apoplast where they were subsequently transferred to the symplast and processed by DCL enzymes into siRNAs (Koch *et al.*, 2016; Biedenkopf *et al.*, 2020). It is probable that cellular internalization of some exRNAs is facilitated by their association with carrier molecules such as RBPs (Numata *et al.*, 2014, 2018). In fact, several studies suggest that, unlike dsRNAs in complex with carrier proteins, naked dsRNAs exogenously applied to leaves are not as effective in silencing endogenous plant genes, while they efficiently silence genes of invading pathogens (Numata *et al.*, 2014; Dalakouras *et al.*, 2018; Brosnan *et al.*, 2021, Preprint; Zhang *et al.*, 2022).

It is also possible that plant exRNAs can act as signaling molecules that do not need to be internalized to exert an effect in recipient cells. Lee *et al.* (2016) showed that pre-infiltrating Arabidopsis leaves with total RNA or rRNA isolated from *P. syringae* elicited plant immune responses similar to that of typical PAMP-triggered immunity (PTI) (Lee *et al.*, 2016). In addition, Niehl *et al.* (2016) showed that PTI responses elicited by application of long dsRNAs are dependent on the plasma membrane-localized pattern recognition co-receptor kinase SERK1. These responses were not dependent on DCLs, indicating that dsRNA-mediated PTI signaling operates independently of the RNAi machinery (Niehl *et al.*, 2016). These findings suggest that extracellular dsRNAs are perceived by a plasma membrane-associated pattern recognition receptor (PRR) that forms a receptor complex with SERK1 to induce PTI signaling (Niehl and Heinlein, 2019). Whether plant exRNAs can be similarly perceived by other organisms remains to be tested.

## Conclusions

The existence of RNAs outside plant cells has been recognized for a long time, but it was not until recently that the biological relevance and importance of this pool of RNAs began to be considered beyond the widely held assumption that they are simply cellular waste. Although much effort has been made to elucidate the packaging and release of RNAs inside EVs, recent work has shown that the majority of exRNAs, including sRNAs, are located outside EVs. How this exRNA is protected against degradation by extracellular RNases is not entirely clear but appears to require association with proteins. In addition, we speculate that extracellular RNases play an important role in shaping the exRNA pool, eliminating many of the RNAs originally released into the apoplast, with post-transcriptional modifications along with RBPs probably playing a significant role in protecting the exRNAs that remain. It should be emphasized that identification of many exRNAs is still challenging due to the technical limitations posed by conventional RNAseq protocols. Yet, the diversity of extracellular RNAs has begun to be elucidated and, to our surprise, it seems to be much more complex than anticipated. Such diversity encourages us to speculate that exRNAs contribute to multiple biological functions, such as cell to cell communication, defense against pathogens, and shaping the plant microbiome.

Plant exRNA research is still in its infancy. and new and exciting knowledge will come to light as this field expands. Indeed, the repertoire of exRNAs in plant species other than Arabidopsis still needs to be characterized, which is critical for identifying exRNAs that are broadly conserved, and thus functionally important. Elucidating how exRNAs reach the apoplast, as well as assessing how they are regulated in response to various stresses will also help us understand what these RNAs are doing ‘outside’ of cells.

**Box 1. Glossary of exRNA types**

Long non-coding RNAs (lncRNA): RNAs longer than 200 nt that are not translated into proteins. The most abundant extracellular lncRNAs described so far are intergenic RNAs (Zand Karimi *et al.*, 2022). These RNAs are transcribed from regions between two protein-coding genes.

Circular RNAs (circRNA): single-stranded, covalently closed RNAs produced via back-splicing or from lariat precursors. The 3ʹ and 5ʹ ends of a circRNA covalently bond together to form a circular RNA molecule. Although the vast majority are expected to be non-coding (ncRNAs), studies in mammals demonstrate that some circRNAs can be translated into proteins (Pamudurti *et al.*, 2017; Yang *et al*., 2017).

rRNAs: non-coding RNAs that constitute the major components of ribosomes. The primary structure of rRNAs is characterized by the presence of intramolecular base pairing, resulting in stem–loop configurations.

tRNAs: non-coding RNAs (76–90 nt) that transfer individual amino acids to ribosomes for assembly into the growing protein during translation.

Small regulatory RNAs: non-coding RNAs (21–24 nt) that play a central role in RNA silencing. sRNAs can be selectively loaded into an ARGONAUTE protein (AGO) to silence target genes via nucleotide base-pairing. According to their biogenesis they can be classified into the following types.

miRNAs: miRNAs are originated from imperfectly paired single-stranded hairpin precursors encoded by *MIR* genes. In miRNAs biogenesis, DICER-LIKE proteins (DCL) cut the hairpin precursors to produce mature miRNAs.

siRNAs: in contrast to miRNAs, siRNAs originate from DCL cleavage of dsRNA molecules that were synthesized from ssRNAs by RNA-dependent RNA polymerases (RdRPs). siRNAs can be further classified into two main categories: heterochromatic siRNAs (hc-siRNAs) and secondary siRNAs, including phased siRNAs (phasiRNAs) and trans-acting siRNAs (tasiRNAs) (Axtell, 2013).

Other small RNAs: 18–34 nt RNAs derived from endonucleolytic processing of diverse RNA species, mainly tRNAs (tRFs and tRNA halves), rRNAs (rRFs), mRNAs, and Pol IV products (Baldrich *et al.*, 2019; Zand Karimi *et al.*, 2022).

Tiny RNAs (tyRNAs): very short RNAs (10–17 nt) with unknown functions. These RNAs are likely to be degradation products derived from multiple sources, including TEs, mRNA, miRNAs, pol IV products, intergenic regions, and rRNAs (Baldrich *et al.*, 2019; Zand Karimi *et al.*, 2022).
